# A Study of the Mechanism of the Chaperone-like Function of an scFv of Human Creatine Kinase by Computer Simulation

**DOI:** 10.1371/journal.pone.0062147

**Published:** 2013-04-24

**Authors:** Jianyu Feng, Hong Guo, Sen Li, Tun Lu

**Affiliations:** 1 College of Biological Science and Engineering, Fuzhou University, Fuzhou, China; 2 College of Mathematics and Computer Science, Fuzhou University, Fuzhou, China; 3 Beijing Key Laboratory of Genetic Engineering Drugs and Biotechnology, College of Life Sciences, Beijing Normal University, Beijing, China; Wake Forest University, United States of America

## Abstract

A new application of antibodies is to use them as macromolecular chaperones. Protein antigens usually have multiple epitopes, thus, there may be a plurality of antibodies binding to one antigen. However, not all antibodies that bind to one antigen could act as a chaperone. Experiments show that some screened anti-human creatine kinase single chain antibodies (scFV) could assist in the folding and stabilizing of the enzyme, while others could not. We built the model of the single chain antibody (scFv-A4) that increased the stability of human creatine kinase (HCK) by the homology modeling method. Epitopes of human creatine kinase were predicted by computer and then the binding of scFv-A4 and HCK was modeled with computer. The calculation results were further combined with the peptide array membrane experiment results to obtain reliable models for the scFv-A4-HCK complex. Based on the above study we gave an explanation about how scFv-A4 could act as a macromolecular chaperone assisting the folding of HCK. This study provides an approach for predicting antigen-antibody binding mode and also a useful theoretical guidance for the study of antibodies' chaperone-like function.

## Introduction

In recent years, a number of human diseases, such as Alzheimer’s, Huntington’s, Parkinson’s, and Creutzfeldt-Jakob’s diseases, were reported to be related to the misfolding and aggregation of proteins [1], [2]. Molecular chaperones are a kind of protein that are capable of assisting nascent peptides in correctly folding to functional proteins by binding to the folding intermediate to avoid kinetic traps, suppressing aggregation of the substrate [3], [4]. Traditional molecular chaperones could be classified according to their molecular weights and sequences to families such as HSP90, HSP70, HSP60 and nucleoplasmin. They have low specificity and react with many kinds of proteins. The low specificity of conventional molecular chaperones enables them to help many house-keeping proteins simultaneously. But a protein-misfolding disease might be caused by only one specie of protein which carries point mutants while most other house-keeping proteins are normal [5]. Thus the use of traditional molecular chaperones as therapeutic molecules for misfolding diseases may have problems such as low efficiency and undesired side-effects. A new field in development is to design or screen specific macromolecules which could be used as chaperones for target proteins, inhibiting their misfolding or coagulation to cure the related protein-misfolding diseases [6]–[8]. Antibodies are the most common macromolecules that can bind specifically to target proteins. Previous researches had shown that some antibodies could excert a chaperone-like function on their antigens [9]. Therefore antibodies with a chaperone-like function were considered as the therapeutic drug candidates for protein misfolding diseases because they only affect mutant proteins, leaving normal proteins intact. In addition, antibodies with a chaperone-like function were helpful research tools for protein folding researches.

Human creatine kinase (HCK) is a protein of important physiological function, which is closely related to intracellular energy operation, muscle contraction and ATP regeneration [10]. According to existing researches in the folding of HCK, the dysfunction of HCK could be a highly possible pathogenic factor of many serious diseases [11], [12]. Our previous studies [13] indicate that HCK expressed in E. coli existed as inclusion bodies. Antibodies produced by using HCK expressed by E. coli as antigen could be used to study the renaturation of inclusion bodies, such as capturing the intermediates during the folding process of HCK to study the structural characteristics of the intermediates. An scFv is a fragment of a conventional antibody which is constructed by connecting the variable domains of the antibody heavy chain and the light chain by a segment of linker peptide. ScFvs with high affinity and specificity to their antigens had been isolated from phage display libraries by many groups [14]–[17]. Schlattner [18] have successfully isolated several scFv clones from a human antibody phage display library that recognize cytosolic BB-CK.

In our previous work [13] several scFvs had been screened out from a phage library using recombinational HCK as antigen. Only one of the scFvs named scFv-A4 has a significant chaperone-like function, preventing the aggregational precipitation of HCK during its folding and accelerating its recovery to nature conformation. In order to comprehend the unique chaperone property of scFv-A4, the binding between scFv-A4 and HCK must be analyzed. The top priority would be identifying the portion of HCK bound by scFv-A4.

Molecular docking by computer has been widely used in the study of the binding mechanism of protein-protein or protein-ligand systems, including themes such as protein-folding mechanism [19], [20], protein-protein surface modification [21], [22], improving the binding between proteins and polypeptides or small molecules [23]–[25]. The number of degrees of freedom of protein-protein interactions was so large that no docking program nowadays can search the conformational space thoroughly even at the classical mechanics level of theory. Therefore, no current software is able to correctly dock two proteins ab initio. One approach to reduce the search space for protein-protein interactions is to predict protein-protein interaction sites first and then use the orientational docking method to test the possibility of the binding. This is the approach used in this work.

As to antigen-antibody binding, the parts of the antibodies bound to antigens were defined as CDR regions (complementarity determining regions) which could be identified by sequence analysis, while the interaction sites on the antigens were called the epitopes. Many bioinformatics software tools had been developed to predict epitopes. B-cell epitopes typically belong to one of the two classes: linear (continuous) epitopes or conformational (discontinuous) epitopes. At present the prediction methods for linear epitopes had been extensively studied and the prediction accuracy had been improved to be as high as 73.37% [26]. Thus linear epitopes prediction programs could be used to predict the potential binding sites of HCK. One technique recently developed to screen potential linear epitopes was the peptide array membrane technique [27]. Peptide fragments covering the whole sequence of a protein antigen were synthesized and spotted on the membrane, the membrane was then treated with antibodies. After washing away the unbound antibodies and labeling the bound antibodies with secondary chromogenic antibodies, the peptide segments of potential epitopes could be identified. Such identified peptides were only potential epitopes because when they were fixed on the membrane their conformation might not be the same as when they were parts of a protein. But we expected more reliable results could be deduced by combining the two methods.

After the potential interaction sites of both interacting proteins had been identified, molecule docking software packages were used to generate 3D models for the scFv-antigen complexes. The quality of the 3D complex models can be used to verify the validity of the previous prediction based on 1D sequence. The chaperone-like function of the antibody was explained by investigating their interactions. The steps of this research were summarized as below. First, the three-dimensional structure of scFv-A4 was constructed by the homology modeling method. Then potential epitopes of HCK were predicted by software tools and the peptide array membrane experiment. The 3D models of scFv-A4-HCK complex were built by orientational docking. Finally, we analyzed the interaction between HCK and scFv-A4 and speculated the mechanism underlying the chaperone-like function of the scFv-A4. This research also provided a new approach for studying antibody-antigen interactions based on the peptide array membrane experiment and bioinformatics software tools.

## Results

### ScFv-A4 Modeling

**Figure 1:**
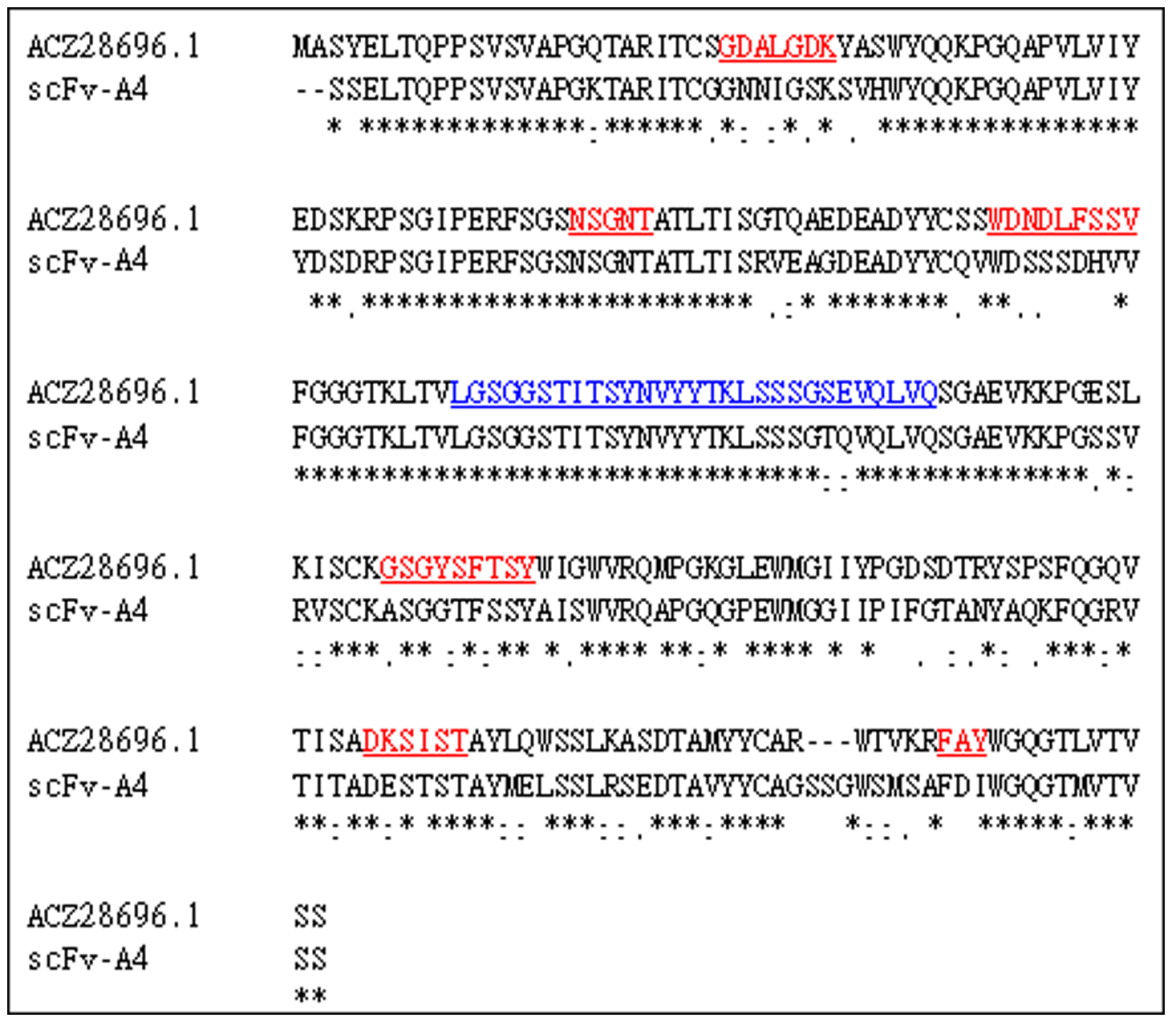
Sequence alignment of scFv-A4 and ACZ28696.1. The ACZ28696.1 CDRs were underlined in red. The light chain and the heavy chain of scFv-A4 had three CDRs respectively. Linker of ACZ28696.1 was underlined in blue. The *symbol indicates same residues between scFv-A4 and ACZ28696.1. The : symbol indicates similar residues between scFV-A4 and ACZ28696.1.The.symbol indicates weakly similar residues between scFv-A4 and ACZ28696.1.

**Figure 2:**
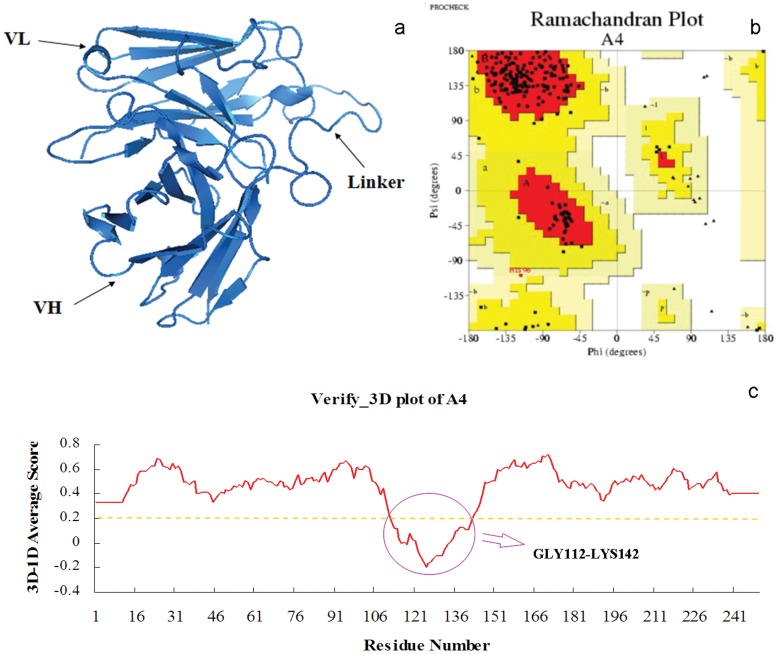
The 3D structure model of scFv-A4. (a) The 3D structure model of scFv-A4 by homology modeling. (b) The Ramachandran plot of scFv-A4 3D model. (c) The Verify_3D plot of scFv-A4 3D model.

The CDRs and the linker of scFv-A4 were inferred from the alignment as shown in Figure 1. The model of the antibody was constructed by the Modeller 9v7 program as shown in Figure 2a. The distributions of Φ–Ψ dihedral angles of the backbone were checked by the Procheck program [28]. A Ramachandran map was used to describe the dihedral angles of the low-energy conformation (Figure 2b). It showed that 87.4% of the residues fell into the best area, 12.1% of the residues into the other license area, 0.5% of the residues into the permitted area, 0.0% of the residues into the not allowed area. Therefore, dihedral angles of the structure of the model were reasonable. For 87.65% residues the 3D–1D compatibility score is greater than 0.2 by Verify-3D [29] (A high score indicated the compatibility of the sequence and the tertiary structure is good). The amino acids with scores less than 0.2 were GLY112-LYS142 which belonged to the linker segment (Figure 2c). Since no template was used for modeling the linker the poorer model result of this area was expected. The above evaluations verified that the three-dimensional model of scFv-A4 was reasonable.

### Peptide Array Membrane Elisa Experiment

**Figure 3:**
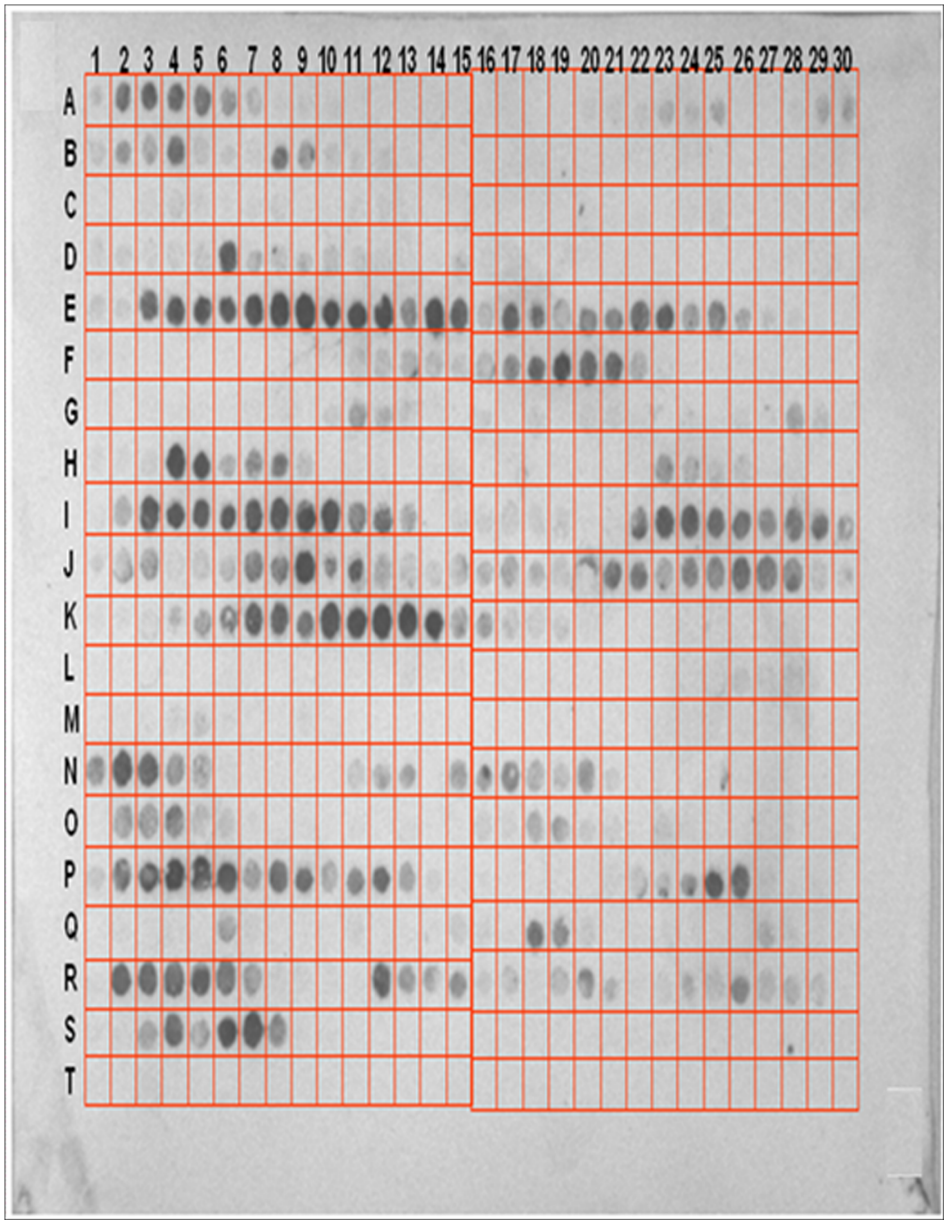
Screening of peptides of HCK binding to scFv-A4 by the peptide array membrane method. The 12 mer peptides from HCK sequence were synthesized on nitrocellulose membrane as individual spots. The membrane was incubated with purified scFv-A4 antibodies in MP buffer overnight. After gentle washing, the peptide-bound antibodies were electro-transferred onto PVDF membrane and detected by western blot.

The peptide array membrane was shown in Figure 3. Each square contained a 12 mer peptide. First set of peptides covered HCK sequence was spotted in squares A1 to M9. Peptides at the spots N9 to T5 were the peptides picked from first set in which first amino acid sequence numbers were odd number; this just served as a repeat experiment. After secondary antibodies and chromogenic substrates were added, six sets of continuous spots were developed. Each set of the continuous spots corresponds to a peptide segment of HCK. Spots A2-A6 correspond to Asn5-Tyr14 of HCK; Spots of E3–E13 correspond to Ser129-Lys138 of HCK; Spots of F17–F21 correspond to Lys170-Met179 of HCK; Spots of I3–I12 correspond to Glu248-Leu257 of HCK; Spots of I22–I29 correspond to Lys265-Asn274 of HCK; Spots of K5–K14 correspond to Thr313-Thr322 of HCK. These six peptides were potential scFv-A4 binding sites on HCK.

### Epitopes Predicted by Ellipro and FBCPred

There were 11 linear epitopes predicted by ElliPro [30] and 15 linear epitopes predicted by FBCpred [26]. These predicted epitopes were potential binding sites for all antibodies. For example, some predicted peptides might be the epitopes for anti-HCK antibodies with no chaperone-like functions. There were a total of eight overlapping epitopes in the two groups of epitopes predicted by both methods. These eight epitopes were shown in Table 1. For these eight peptides, four peptides fell in the N-terminal domain, one peptide fell in the linker area and three fell in the C-terminal domain. Comparing to the peptide array membrane experiment results, there were two overlapping sequences, LYS170-MET179 and LYS265-ASN274, which were positive in both methods.

**Table 1:** B-cell Linear Epitopes of Human CK predicted by both of ElliPro and FBCPred.

	ElliPro(*score*)	FBCPred(*score*)	Overlap Region
1	8–27(*0.874*)	15–28(*1.0*)	15–27
2	34–49(*0.778*)	37–50(*1.0*)	37–49
3	61–69(*0.589*)	58–71(*0.991*)	61–69
4	110–125(*0.626*)	117–130(*0.944*)	117–125
5	152–191(*0.724*)	174–187(*0.994*)	174–187
6	219–224(*0.582*)	219–232(*0.887*)	219–224
7	262–270(*0.703*)	264–287(*0.756*)	264–270
8	358–381(*0.826*)	357–370(*0.999*)	358–370

Each peptide epitope was noted by its sequence region on HCK. There were total eight epitopes predicted by both software and their overlapping region was listed at the right column.

### Binding Models Built by Docking

**Figure 4:**
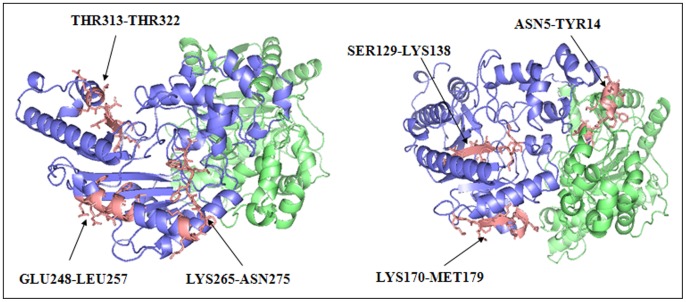
Positions of the peptides screened out by the peptide array membrane method on the HCK 3D structure. The six experimental positive peptides were colored in salmon and represented by using the stick model. As shown in the picture, ASN5-TYR14 is at the position of dimerization interface of the two monomers; SER129-LYS138 is completely embedded inside the structure; GLU248-LEU257 is in a dent of the surface; LYS265-ASN274 which contains V8 endoproteinase fragment was also in a dent of the structure; LYS170-MET179 is located at the bottom of the structure; THR313-THR322 is located in the largest groove of the structure.

An orientational docking of scFv-A4 and HCK epitopes was carried out by ZDOCK [31] docking server. If we combined the results from peptide array membrane experiment and bioinformatics predictions, we could reduce the workload by only docking the two overlapping peptides. For experimental completeness, we did the orientational docking for all of the six peptides screened out by the peptide array experiment. ZDOCK did not generate any reasonable models for four of the six peptides (SER129-LYS138, GLU248-LEU257, THR313-THR322, ASN5-TYR14). It was interesting because it agreed with the bioinformatics prediction that they were not epitopes. To look into the details of the six peptides, we investigated their structure feature. As shown in Figure 4, the first three peptides were embedded inside the HCK monomers, thus they were not accessible by scFv-A4. For peptide ASN5-TYR14, it was buried between the dimerization interfaces of the two HCK monomers therefore it was also inaccessible for scFv-A4. Only the rest two peptides (LYS170-MET179 and LYS265-ASN274 could be docked to scFv-A4 by ZDOCK Server and two complex models were obtained.

**Figure 5:**
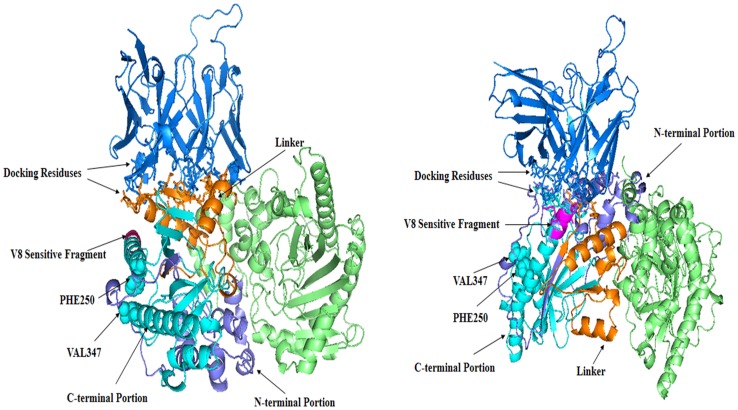
The scFv-A4-HCK complexes. Model I is shown at left. Model II is shown at right.ScFv-A4 is colored in marine. HCK is shown as dimmer. One HCK monomer is colored in lime. The N-terminal of the other HCK monomer is colored in slate and C-terminal in cyan, the linker is colored in orange. The V8 endoproteinase sensitive fragment is colored in magentas. The binding residues are represented by using the stick model. The PHE250, VAL347 are represented using the sphere model.

Because ZDOCK used rigid body approximation, to obtain a more accurate protein complex structure, complex models generated by ZDOCK were redocked by RosettaDock [32]. which implemented the flexible docking method and came with a more accurate scoring function. RosettaDock generated 1000 new complex models for each of the two epitopes respectively. The output results were ranked by docking scores. We discarded models in which the binding sites of the antibody were outside the CDR regions and picked the best complex models by the best rank scores. Two final complex models for the two epitopes were named as complex-I and complex-II respectively and shown in Figure 5.

## Discussion

Antibodies and their functional derivatives such as scFv and Fab are known for their specific binding and have already been used in protein folding researches [9]. Antibodies with a chaperone-like function had the potential to be used as therapeutic agents for misfolding diseases. More researches need to be done to elucidate the mechanism of antibodies’ chaperone-like function. A protein antigen usually carries more than one epitope, therefore an antigen can interact with several different types of antibodies. Obviously the different types of antibodies have different impacts on the antigen’s properties. We had previously screened out several scFvs for HCK and one of them named scFv-A4 showed strong chaperone-like activity. To elucidate why scFv-A4 had the chaperone-like function, we combined computer modeling and peptide array membrane technique to study the interactions between scFv-A4 and HCK.

Firstly, bioinformatics software tools were used to predict the interacting sites between scFv-A4 and HCK and then the results were combined with the peptide array membrane experiment results to build the 3D models of the binding complex. The prediction of protein-protein interactions by computer is still a challenging subject, and it is inappropriate to rely entirely on the energy scoring function to filter the results directly. Thus, we used the orientational docking method followed by redocking, the cumulated results were then sorted by the scoring function to get reliable protein-protein binding models. First the binding sites of the antigen and the antibody were predicted respectively. After that the two proteins were orientationally docked according to the predicted binding sites. Sequence based epitope prediction was still difficult especially for the conformational epitopes. We combined the prediction results of two different software tools, and only those epitopes reported by both methods were considered as potential epitopes. The accuracy of each method was 73% (FBCPred) and 84% (Ellipro) respectively, so the accuracy of the prediction of the two methods combined could reach 95%. The number of predicted epitopes was further reduced by comparing them with the peptide array membrane experiment results. The epitopes we studied were linear epitopes, and for linear peptide epitopes, the binding solely depends on amino acid sequences. If an antibody could bind to a linear peptide epitope, it will bind to the peptide irrespective of whether the peptide was on the protein, on the folding intermediate or on the membrane. Consequently only the epitopes which were positive in peptide array membrane experiment were selected for further modeling. Two antigen-antibody model complexes were screened out as the final result.

There are two major entry points for scFv-A4 to exert its chaperone-like function on HCK, on the HCK folding intermediate or on the folded HCK. Yan et al found that the C-terminal domain of HCK was more likely to be the binding site during rabbit muscle CK (RMCK) aggregation [33] and the linker played a crucial role in RMCK folding [34], [35]. After further investigate, they found that the linker act as a lid to protect the hydrophobic side chains of PHE250 and VAL347 against exposure to solvent [36]. In complex model-I, scFv-A4 bound to the opposite site of HCK thus has not impact on these residues (Figure 5a). In complex model-II, the binding position of scFV-A4 was close to the two hydrophobic residues and actually formed a bulky protrusion (Figure 5b), therefore for complex model-II, scFv-A4 bound to HCK would hinder the aggregation of HCK by steric effects.Webb [37] found that the C-terminal portion of the chick CK (GLY215 to LYS380) was much more resistant to digestion than the N-terminal portion (PRO1 to GLY133), and the middle of the HCK sequence (ARG134 to ARG214) was most sensitive to proteolysis. They proposed that the folding intermediate consisted of a folded C-terminal domain and a partially folded N-terminal domain separated by an unfolded central linker. Gross et al.'s studies suggested that the C-terminal fragment (168–380) of mitochondrial CK represented an autonomous folding unit, and that the folding of the C-terminal domain might precede the conformational stabilization of the N-terminal moiety (1–167) in vivo [38]. The binding site of complex model-I was located between these two terminal domains, which indicated that in complex model-I, scFv-A4 would not significantly interfere with the major folding path ways of the two domains. It may stabilize the folded C-terminal domain and assist in the folding of the N-terminal domain. There was a V8 endoproteinase sensitive fragment in the chick CK intermediate. When the chick CK had been resurrected, this fragment resisted to the proteinase, since it became less exposed in the folded kinase [39]. Therefore, this fragment can be regarded as a characteristic part of the creatine kinase intermediate. This fragment identified by V8 endoproteinase was IFKKAG [37], the corresponding sequence of HCK was ILE263-GLY268. It is interesting that in complex model-II, scFv-A4 also bound to this segment which indicated that scFv-A4 could bind with the intermediate and participate in HCK folding or resurrecting process. To summarize, in complex model-II scFv-A4 can exhibit a chaperone-like function either by hindering the aggregation by the steric effects or by assisting the folding of HCK and stabilizing the folded conformation. In complex model-I, scFv-A4 exhibits chaperone-like function by assisting the folding of HCK and stabilizing the whole structure.

In this study we combined computer modeling and the peptide array membrane method to investigate the interaction between scFv-A4 and HCK. The mechanisms underlying scFv-A4’s chaperone-like function were discussed. The traditional method of studying antigen-antibody interactions is by constructing many antigen mutants and observing the changes of the binding activity. The whole process is tedious and time consuming. Epitope-scanning by Peptide array membrane method has been made cheap and fast nowadays, our work demonstrated that the combination of the peptide array membrane technique with bioinfomatics methods could be a more efficient approach for epitope mapping. For the two complex models we proposed, further study can be done to verify the models by engineering point mutants on the predicted epitopes of HCK and investigating the change of the system’s behaviors.

## Materials and Methods

### Homology Modeling of scFv-A4

Homology modeling in our study was done in the following three steps: (1) Speculating the linker, the light and the heavy chain segments of the antibody. The sequence of scFv-A4 was used to do a BLAST search in the Non-redundant protein sequences database of NCBI. A scFv sequence (ID: ACZ28696.1) [40] which had the highest score and the lowest E value was picked out to be used as reference. The sequences of ACZ28696.1 and scFv-A4 were aligned by Clustalx1.81 [41] and each area of the scFv-A4 sequence was appointed by comparing the sequence with ACZ28696.1. (2) Homology modeling templates preparation. Since linker peptides of the single chain antibody were often absent in the PDB database [42], the sequence of the linker peptide would be excluded when aligned with the template antibody. The sequence of scFv-A4 without linker was used to search the PDB database. The light chain and the heavy chain were processed separately. We obtained a template (2DD8 [43]) with a higher identity which was a Fab antibody fragment. The sequence identity of the light chain of the Fab was 100%, while that of the heavy chain was 93%. Both chains of the Fab were used as templates for building the 3D model of scFv-A4 by Modeller 9v7 [44]. (3) Optimization of the model. Since the linker was modeled without an appropriate template, optimization work was focused on the linker. The DOPE-based loop modeling protocol [44] was used to optimize the linker structure.

### Determination of Antibody CDR Region

The CDRs are the major region of the antibodies that bind to antigens. Since the CDRs of the scFv ACZ28696.1 was known, the CDRs of scFv-A4 was inferred from the alignment result of the two sequences (Figure 1). The resulted CDR assignment was checked to be complied with the Kabat numbering rules [45].

### Building the 3D Model of HCK

The crystal structure of HCK was available in PDB file 1I0E [46]. HCK is a dimmer in nature but the 1I0E only contains a monomer and the structure of two peptide fragments, MET1-HIS7 and GLY323-GLY331, was missing. Thus we use another CK structure, the 1U6R for rabbit CK in PDB as the homology modeling template. The sequence identity between rabbit CK and HCK is 96% and the 1U6R file contains the structure of the two missing fragments peptides of the 1I0E file.

### Epitopes Prediction by Software Tools

We used FBCPred [26] based on the machine learning method and ElliPro [30] based on the 3D structure for epitope prediction. An epitope predicted by both methods would be regarded as a potential epitope. As for FBCPred, default parameters were adopted for linear epitope prediction, the length of the linear epitopes was set to 14, and the specificity was 75% as default. No parameter was needed for ElliPro.

### Screening of Epitopes by the Peptide Array Membrane Method

12 mer peptides from the HCK sequence were synthesized on nitrocellulose membrane as individual spots. Peptide sequences were started from HCK N-terminal and each subsequent peptides shift one position to the right. A set of such peptides completely sampled the whole HCK sequence and formed an array on the membrane. The peptide array membrane was incubated with purified scFv-A4 antibodies in the MP buffer (30 mM Tris-HCl, pH 7.6, 170 mM NaCl, 6.4 mM KCl, 0.05% Tween 20, 5% sucrose) overnight at 4°C with gentle shaking. Unbound antibodies were removed with TBS (4°C) and the antibodies bound were electro-transferred onto a PVDF membrane using a semi-dry blotter (0.8 mA/cm2, three hours). The membrane was blocked with 5% non-fat dry milk for 2 h and incubated with the primary antibody (anti-V5 antibody) overnight at 4°C. After being washed with TBS buffer for three times, the membrane was incubated with the alkaline phosphatase-conjugated secondary antibodies for 1 hour and developed using the enhanced chemiluminescence method.

### Molecular Docking

The ZODCK Server was used to dock scFv-A4 to HCK. The ZDOCK Server allowed user to pick contact and blocking residues of the proteins for docking. The HCK residues of the predicted epitopes were chosen as contact residues for docking. There were six linear peptides exhibiting positive reactivity with scFv-A4 in the peptide array membrane experiment and the corresponding portions of HCK were designated as the orientational docking sites of HCK. No blocking residues of HCK were selected during docking, but for scFv-A4, residues of the linker segment were selected as the blocking residues. RosettaDock was used to optimize the models build by ZDOCK because it implemented the flexible docking method and had a scoring function of better performance. The perturbation docking method of the RosettaDock program was applied and the three default perturbation parameters were used. One thousand models were produced for each selected ZDOCK model complex.
